# O-Glycosylation in Cell Wall Proteins in *Scedosporium prolificans* Is Critical for Phagocytosis and Inflammatory Cytokines Production by Macrophages

**DOI:** 10.1371/journal.pone.0123189

**Published:** 2015-04-14

**Authors:** Mariana I. D. S. Xisto, Vera C. B. Bittencourt, Livia Cristina Liporagi-Lopes, Rosa M. T. Haido, Morena S. A. Mendonça, Guilherme Sassaki, Rodrigo T. Figueiredo, Maria Teresa V. Romanos, Eliana Barreto-Bergter

**Affiliations:** 1 Departamento de Microbiologia Geral, Instituto de Microbiologia Paulo de Góes, UFRJ, Rio de Janeiro, Rio de Janeiro, Brazil; 2 Departamento de Microbiologia e Parasitologia, Instituto Biomédico, UNIRIO, Rio de Janeiro, Rio de Janeiro, Brazil; 3 Departamento de Análises Clínicas e Toxicológicas, Faculdade de Farmácia, UFRJ, Rio de Janeiro, Rio de Janeiro, Brazil; 4 Instituto de Ciências Biomédicas, UFRJ, Rio de Janeiro, Rio de Janeiro, Brazil; 5 Departamento de Bioquímica e Biologia Molecular, UFRP, Curitiba, Paraná, Brazil; 6 Campus de Xerém, Instituto de Ciências Biomédicas, UFRJ, Rio de Janeiro, Rio de Janeiro, Brazil; 7 Departamento de Virologia, Instituto de Microbiologia Paulo de Góes, UFRJ, Rio de Janeiro, Rio de Janeiro, Brazil; Institut National de la Santé et de la Recherche Médicale (INSERM), FRANCE

## Abstract

In this study, we analyze the importance of *O*-linked oligosaccharides present in peptidorhamnomannan (PRM) from the cell wall of the fungus *Scedosporium prolificans* for recognition and phagocytosis of conidia by macrophages. Adding PRM led to a dose-dependent inhibition of conidia phagocytosis, whereas de-*O*-glycosylated PRM did not show any effect. PRM induced the release of macrophage-derived antimicrobial compounds. However, *O*-linked oligosaccharides do not appear to be required for such induction. The effect of PRM on conidia-induced macrophage killing was examined using latex beads coated with PRM or de-*O*-glycosylated PRM. A decrease in macrophage viability similar to that caused by conidia was detected. However, macrophage killing was unaffected when beads coated with de-*O*-glycosylated PRM were used, indicating the toxic effect of *O*-linked oligosaccharides on macrophages. In addition, PRM triggered TNF-α release by macrophages. Chemical removal of *O*-linked oligosaccharides from PRM abolished cytokine induction, suggesting that the *O*-linked oligosaccharidic chains are important moieties involved in inflammatory responses through the induction of TNF-α secretion. In summary, we show that *O*-glycosylation plays a role in the recognition and uptake of *S*. *prolificans* by macrophages, killing of macrophages and production of pro- inflammatory cytokines.

## Introduction

*Scedosporium prolificans* is an opportunistic pathogen, responsible for serious infections in immunocompetent as well as immunocompromised patients, due to its high virulence and antifungal multidrug resistance [1, 2].

Cell wall surface glycoconjugates from the *Scedosporium*/*Pseudallescheria boydii* complex are thought to be the first point of contact between the fungus and cells of the innate immune system [3, 4]. *N*- and *O*-linked peptidorhamnomannans are major pathogen-associated molecular patterns and along with α-glucans, play important roles in triggering host innate immunity [3, 5, 6].

Rhamnose-containing structures appear to be immunodominant epitopes in the peptidorhamnomannans isolated from mycelia of *P*. *boydii*, *S*. *prolificans* and *S*. *apiospermum*, particularly if they are present as (1→3)-linked α-Rha*p* side chain units [7–10]. Antibodies recognizing this structure may, therefore, recognize both the *N*-linked high molecular weight polysaccharides and the *O*-linked oligosaccharides in the glycocomplexes.

A peptidorhamnomannan (PRM) that consists of a peptide chain substituted by *O*- and *N*-linked glycans was also isolated from the conidia cell wall of *S*. *prolificans* and its structure elucidated using methylation analysis and ^13^C-nuclear magnetic resonance [11]. Although the oligosaccharides obtained from -elimination of PRM from *S*. *prolificans* conidia and from *S*. *prolificans* mycelium are structurally different, they have the x-Rha*p*-(1→3)-α-Man*p*-(1→2)-α-Man*p*- group in common, which is conserved in PRM from the mycelium of *P*. *boydii* and *S*. *apiospermum*, as well as *Sporothrix schenckii* [5, 11–13]. In this study, we analyzed how the glycosylation of *S*. *prolificans* proteins influences the recognition and uptake of *S*. *prolificans* by macrophages, as well as its role in killing macrophages and promoting the production of pro-inflammatory cytokines.

## Materials and Methods

### Microorganism growth conditions

A culture of *S*. *prolificans* was supplied by Dr. J. Guarro, Unitat de Microbiologia, Facultat de Medicina e Institut d`Estudis Avançats, Réus, Spain. It was grown in Erlenmeyer flasks containing 200 ml of modified Sabouraud medium, consisting of (g/l) peptone, 10; yeast extract, 5; glucose, 40. Cultures were incubated at room temperature for 7 days with shaking (pre-inoculum). Conidia were grown on Petri plates containing modified Sabouraud medium at room temperature. After 7 days, conidia were obtained by washing the plate surface with phosphate-buffered saline and hyphal fragments and debris were removed by filtration through gauze.

### Mice

Female Balb/C (4–8 weeks years-old) mice were obtained from the Universidade Federal do Rio de Janeiro Breeding Unit (Rio de Janeiro, Brazil). The animals were maintained at constant temperature (25°C) with free access to chow and water in a room with a 12-h light/dark cycle. The experiments were approved by the Institutional Animal Welfare Committee of the Federal University of Rio de Janeiro.

### Extraction of peptidopolysaccharides (PRMs) from conidia

In a typical experiment, conidia were extracted with 0.05 M phosphate buffer, pH 7.2 at 100°C for 2 h, and the mixture was then dialyzed. The centrifugation of retained material provided a supernatant, which was evaporated to a small volume and freeze-dried to give crude glycoprotein (541 mg). An aq. solution was then dialyzed to give retained material (119 mg).

### Fractionation of the PRM

Peptidopolysaccharides were purified by hexadecyltrimethylammonium bromide (Cetavlon, Merck, Darmstadt, Germany) fractionation. The mother liquors from Cetavlon precipitation were adjusted to pH 8.8 in the presence of borate and the resulting precipitates recovered by centrifugation to give the major PRM fraction. The PRM was dialyzed against distilled water and freeze-dried [8].

### Isolation of de-O-PRM

PRM was submitted to β-elimination under reducing conditions [5, 14] to release the *O*-linked oligosaccharides. The solution was neutralized (HOAc) and dialyzed, which allowed the retention of peptide moiety still glycosylated with sugar chains (de-*O*-PRM) that were resistant to mild alkali pretreatment.

### Nuclear magnetic resonance

Two-dimensional homo- and heteronuclear ^1^H/^13^C correlation experiments (HSQC) were obtained using a 600 MHz Bruker Avance III spectrometer with a 5 mm inverse probe (QXI). The analyses were performed at 50 or 70°C in D_2_O, and the chemical shifts were expressed in δ ppm relative to external standard of acetone at δ 30.2 (^13^C) and 2.22 (^1^H).

### Rabbit immune sera

White male rabbits were inoculated with freeze-dried whole cells of *S*. *prolificans* (2 mg/ml dry weight) emulsified in an equal volume of complete Freund’s adjuvant; 1 ml of emulsion was injected intradermally at weekly intervals for 3 weeks [15]. Then, throughout a one-week period, the same concentration was used in three intravenous injections at 2 days intervals. The hyperimmune serum obtained was used in flow cytometry and immunofluorescence experiments. Pre-immune serum was taken as a control.

### Flow cytometry

Conidia (8x10^6^ cells) were fixed in 1% paraformaldehyde in PBS. After being washed 3 times with PBS, nonspecific sites were then blocked by incubation with blocking buffer (PBS—3% BSA) for a period of 1 h at 37°C. After this time, the conidia were incubated with whole cell antiserum to *S*. *prolificans* (1:50) inhibited or not with conidia-derived PRM (100 μg/ml) for 1 h at 37°C in PBS-BSA with subsequent washing in PBS (3 times). After this, other incubation was conducted with anti-rabbit immunoglobulin (conjugated with Alexa) in PBS-BSA for 1 h at 37°C. The conidia were then washed thoroughly with PBS and resuspended in the same buffer, and analyzed on a FACSCalibur flow cytometer (Becton Dickinson). Data from each experiment were analyzed using "Windows Multiple Document Interface Flow Cytometry Application (WinMDI) version 2.8 software." Controls using only the secondary antibody were used to exclude the possibility of nonspecific binding of these antibodies. A control set consisting of spores only was used for the detection of possible intrinsic fluorescence of the samples.

### Immunolocalization of PRM on the surface of conidia—immunofluorescence

Freshly harvested conidia were attached to coverslips coated with poly-L-lysine and then fixed in 1% paraformaldehyde in PBS for 1 h. After being washed 3 times with PBS, Nonspecific sites were then blocked by incubation with blocking buffer (PBS-1% BSA) for a period of 1 h at 37°C. Then, the conidia were incubated with whole cell antiserum to *S*. *prolificans* (at 1:50 dilution) inhibited or not with conidia-derived PRM (100 μg/ml) overnight at 4°C. After washes, anti-rabbit IgG conjugated with AlexaFluor (1/200) were added and incubated "overnight" at 4°C. To mount the blades was used n-propyl gallate (VETEC) (to preserve the sample) and coverslips were sealed with nail polish. The recognition molecule of the antibody, PRM, was visualized through Axioplan fluorescence microscopy.

### Phagocytic assay

Elicited peritoneal macrophages were obtained by the intraperitoneal instillation of 2 ml of 3% sterile thioglycollate. After 3 days, mice were sacrificed, and peritoneal macrophages were harvested and washed with RPMI 1640 and plated. Elicited macrophages (5x10^5^ cells/well) were cultured over round glass coverslips (13 mm) in 24-well flat bottom microtest plates. Adherent monolayers were challenged with 500 μl of live conidia suspensions containing 2.5x10^6^ cells/well. After incubation at 37°C in 5% CO_2_ for 1h in RPMI 1640 medium, the cells were rinsed with RPMI medium for removal of non-internalized conidia. The preparations were fixed in Bouin’s fixative and stained with Giemsa. The influence of intact and de-*O*-glycosylated PRM on conidia phagocytosis was evaluated by adding different concentrations of the glycoprotein (12.5 and 25 μg/ml) 30 min before the addition of conidia. To determine the phagocytic indices (PIs), 200 cells were counted and the percentage of cells that ingested at least one particle was multiplied by the mean number of internalized particles [3].

### Macrophages viability assay

#### Passive adsorption

Intact and de-*O*-glycosylated PRM-coated latex beads were prepared by passive adsorption of intact and de-*O*-glycosylated PRM from conidia of *S*. *prolificans* on the bead surface. Approximately 2x10^9^ polystyrene latex beads (Sigma-Aldrich), 1.1 μm in diameter, were washed twice with centrifuge steps at 10,000*g* for 10 min in 0.1 M of carbonate-bicarbonate buffer, pH 9.6, and incubated with 500 μg of intact and de-*O*-glycosylated PRM from *S*. *prolificans*-derived conidia in 500 ml of the carbonate-bicarbonate buffer for 1 h at 37°C. The beads were then washed twice with HBSS (Invitrogen), incubated in 5% BSA for 2 h at 37°C to block nonspecific binding sites, washed again, resuspended in HBSS, and stored at 4°C before use [16].

#### Macrophage killing assay

The macrophage killing assay was conducted under the same conditions described above for the phagocytosis assay. After removal of excess unbound *S*. *prolificans* and/or intact and de-*O*-glycosylated PRM-coated latex beads (ratio 5:1) by rigorous washing with PBS, the killing of macrophages was assessed by trypan blue exclusion. Cells were incubated with 150 μl of Trypan blue (Sigma) and 150 μl of PBS for 2 min and removed by lightly washing twice with PBS. The cells were then fixed with 3% paraformaldehyde and counted under an inverted light microscope (Nikon Eclipse TE2000-U microscope with a 40x objective) to ascertain the percentage of macrophages killed. Data were obtained in triplicate from at least three separate experiments by analyzing at least 200 macrophages per well [17].

#### Neutral red dye-uptake method

Macrophages were plated in 96-well plates. PRM and de-*O*-glycosylated PRM were added to the wells at concentrations of 200 μg/ml, 100 μg/ml, 50 μg/ml, 25 μg/ml, 12.5 μg/ml, 6.2 μg/ml and 3.1 μg/ml. After 24 h, the cytotoxic effect on macrophages was analyzed by the neutral red technique. The technique consists of the incorporation of neutral red dye (0.01%) by living cells at 37° C for 3 hours. Then, the dye was discarded and the cell monolayer washed with PBS. After washing, 100 μl of 4% formaldehyde in PBS was added for 10 minutes. Then, a mixture of acetic acid (1%) and methanol (50%) was used for disrupting the cells to release and solubilize the dye. After 20 minutes, the reading was taken at 492 nm in a spectrophotometer.

### Macrophage effector functions

#### Measurement of nitric oxide release by macrophages

Peritoneal macrophages were plated at 2x10^5^ cells/well in 96-well polystyrene tissue-culture plates. Heat-killed conidia (ratio 5:1), intact and de-*O*-glycosylated PRM (12.5 and 25 μg/ml), or LPS (O111:B4) were incubated with macrophages for 24 h at 37°C in 5% CO_2_. After 24 h of incubation, the supernatants were collected. Nitric oxide levels were measured using a commercial Griess reagent kit (Promega) after 24 h [10].

#### Cytokine assays

Elicited peritoneal macrophages were obtained by the intraperitoneal instillation of 2 ml of 3% sterile thioglycollate. After 3 days, mice were sacrificed and the peritoneal macrophages were harvested, washed with RPMI-1640, and plated at a density of 2x10^5^ cells/well in a 96-well plate. The plate was incubated for 1 h at 37°C in 5% of CO_2_. Non-adherent cells were removed by washing with RPMI-1640. Adherent cells were stimulated for 18 h, in RPMI medium, with the intact and de-*O*-glycosylated PRM, heat-killed conidia (ratio 5:1) or LPS (O111:B4). After this period the supernatant was recovered for TNF-α and IL-10 determination by ELISA according to the manufacturer’s instructions. Polymixin B (10 μg/ml) was added 5 min before the addition of the stimulus, to rule out the possibility that the stimulating activity was due to contaminating lipopolysaccharides.

After incubating for 18 h, supernatants were harvested, centrifuged at 12 000 rpm for 10 min to remove cell debris and stored in cryogenic vials at -80°C. In the supernatants obtained, the concentration of TNF-α and IL-10 was measured by ELISA (BD OptEIA, Mouse TNF-α and IL-10 ELISA Set) according to the guidelines of the manufacturer.

### Statistical analysis

Statistical analyses were performed using GraphPad Prism version 5.00 for Windows (GraphPad Software, San Diego CA). Unless otherwise noted, a one-way analysis of variance using a Kruskall-Wallis nonparametric test was used to compare the differences between groups, and individual comparisons of groups were conducted using a Bonferroni posttest. A t-test was used to compare the number of CFU for different groups. A 90–95% confidence interval was determined in all experiments.

## Results

### Analysis of S. prolificans N-linked PRM (de-O-PRM)

**Fig 1:**
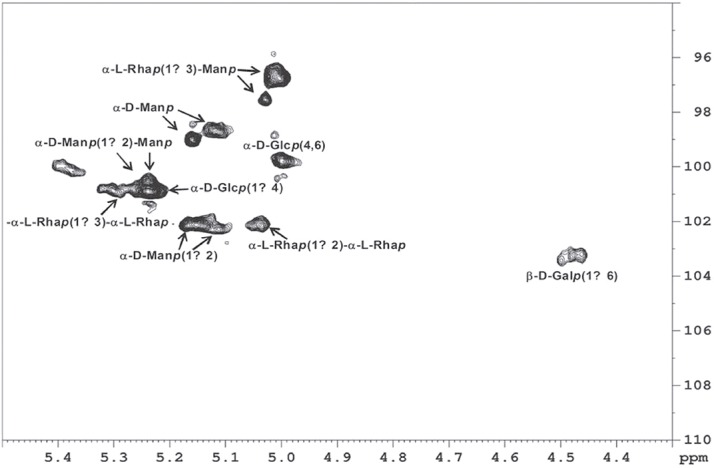
Partial 2D HSQC NMR: NMR spectrum of the anomeric region from *N*-glycan of PRM (rhamnomannoprotein).

The de-*O-*PRM fraction corresponding to the peptide moiety still glycosylated with sugar chains that were resistant to mild alkali treatment was analyzed by NMR. The structure of de-*O*-PRM was confirmed by ^1^H and ^13^C NMR analysis, based on HSQC fingerprints (**Fig 1**). The main signals summarized in **Table 1** were assigned according to their correlation among the NMR spectra and to literature values of similar glycans. The anomeric region (C1/H1) contained signals at 102.53/5.51, 101.14/5.64, 99.25/5.56 and 98.92/5.52 consistent with the presence of α-mannopyranose 2-*O*-substituted and non-reducing end units, respectively [8]. The signal (C1/H1) at 101.08/5.70 showed the presence of α- L-Rha*p* (1→3)-α-L-Rha*p* and the C1/H1 signal at 102.2/5.05 indicates the presence of a terminal α- L-Rha*p* (1→2)-α-L-Rha*p*. The high field signals of C1/H1 at 97.79/5.43 and 97.02/5.41 could be attributed to α- L-Rha*p* (1→3)-Man*p*. [8]. A signal at 103.6/4.43 in its HSQC spectrum indicated the presence of β-Gal*p* units and present in the *O*-linked oligosaccharides from conidia and mycelium of *S*. *prolificans* PRM [8, 11]. The edited HSQC spectrum also showed typical signals of α-glucan [3]. The signal (C1/H1) of (1→4)-linked α-D-Glc*p* was observed at 101.2/5.21. The 4, 6-di-*O*-substitution of the α-D-Glc*p* residues was confirmed by the presence of signals at 100.0/5.01. The anomeric region lacks one signal at δ 101.8 arising from 2-*O*-methyl rhamnose capping group, present in the *S*. *prolificans O*-linked oligosaccharides [11]

**Table 1:** ^1^H and ^13^C NMR chemical shifts of the *S*. *prolificans N*-glycan.

			
Units	^13^C sign *	^1^H sign*	References
C1/H1 of β-D-Galp- (1→6)-	103.3	4.47	a
C1/H1 of α-D-Manp-(1→	99.1	5.13	b
C1/H1 of α-D-Manp-(1→	98.8	5.12	b
C1/H1 of α-D-Manp-(1→2)-	102.4	5.11	b
C1/H1 of α-D-Manp-(1→2)-	102.1	5.15	b
C1/H1 of α-D-Manp-(1→2)- Manp-	100.8	5.24	a, b
C1/H1 of α-L-Rhap (1→3)-Manp-	97.3	5.04	a, c
C1/H1 of α-L-Rhap (1→3)-Manp -	98.5	5.02	a, c
C1/H1 of α-L-Rhap (1→3)-L-Rhap-	101.0	5.30	a, c
C1/H1 of α-L-Rhap (1→2)-L-Rhap-	102.2	5.05	d
C1/H1 of α-D-Glcp-(1→4)-	101.2	5.21	b, e
C1/H1 of α-D-Glcp-(4,6)-	100.0	5.01	b, e

*The chemical shifts are expressed as ppm (δ)

^a^Barreto-Bergter *et al*., 2008 [8]

^b^Smiderle *et al*., 2013 [43]

^c^Gorin *et al*., 2010; Figueiredo *et al*., 2010 [11, 38]

^d^Mendonça *et al*., 1976 [44]

^e^Bittencourt *et al*., 2006 [3]

### Evidence of PRM exposition on the conidia surface

**Fig 2:**
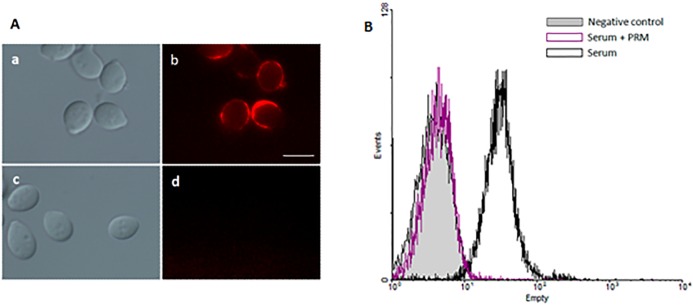
PRM is on the fungal cell surface. Immune serum was able to bind to *S*. *prolificans* conidial forms (**Aa and Ab**), but pre-incubation with soluble pRM was able to inhibit the binding between *S*. *prolificans* conidia cells and immune serum (**Ac and Ad**), as observed by immunofluorescence microcopy. A similar data was observed by flow cytometry showing that conidia fluorescence is practically abolished when serum is pre-treated with PRM (**B**). Bar: 10 μm.

In order to verify if PRM was exposed on conidia surfaces, rabbit immune sera against whole *S*. *prolificans* cells was employed in immunofluorescence experiments. As demonstrated by fluorescence microscopy, the immune serum was able to recognize conidial forms (**Fig 2A**), the sera with soluble PRM was inhibited in its binding to conidia (**Fig 2A**). Consistent with this, flow cytometry showed that conidia fluorescence is practically abolished when serum is pre-treated with PRM, confirming the expression of PRM on the surface of conidial forms (**Fig 2B**).

### Involvement of PRM on phagocytosis of S. prolificans by macrophages

**Fig 3:**
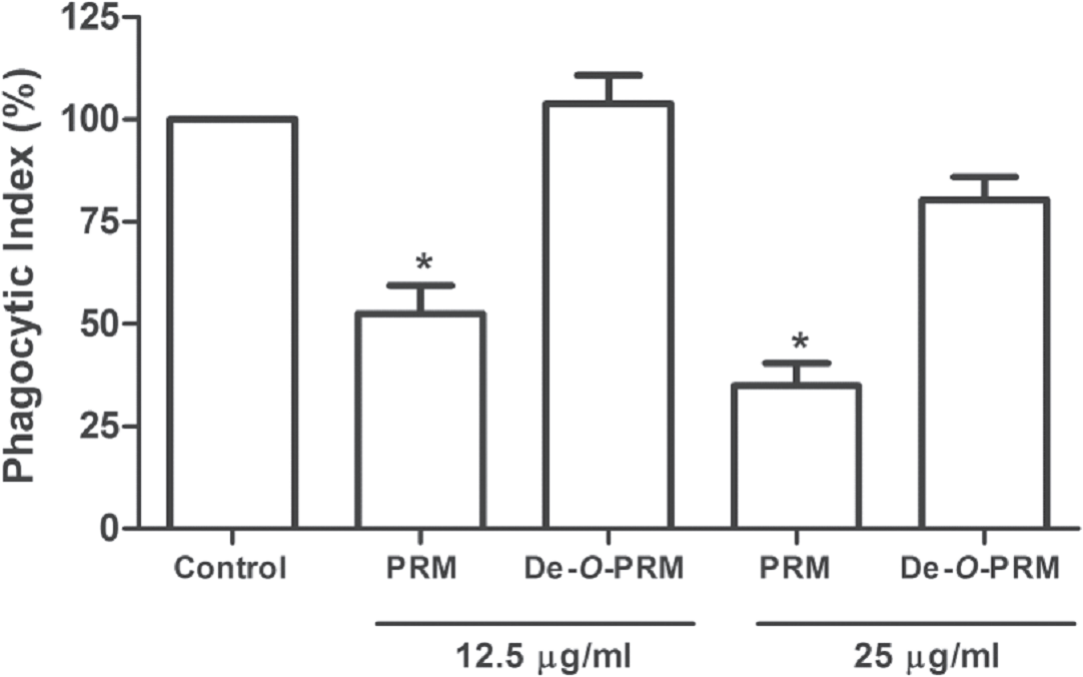
Phagocytosis inhibition assay between *S*. *prolificans* conidia and peritoneal macrophages by intact and de-*O-*glycosylated PRM. The macrophages were pre-treated or not (control) for 30 min with two different concentrations of intact and de-*O-*glycosylated PRM (12.5 and 25 μg/ml), before interacting with conidial cells for 1 h. The phagocytic index values represent the mean ± S.D. of three independent experiments performed in triplicate. Asterisks denote values significantly different from control (* P < 0.05).

To evaluate whether PRM and its *O*-linked oligosaccharides are involved in *S*. *prolificans* uptake, macrophages were treated with 12.5 and 25 μg/mL PRM and de-*O*-glycosylated PRM before interaction with conidia for 1 h. The conidia to macrophage ratio was adjusted to 5:1. The addition of PRM led to the inhibition of conidia phagocytosis, whereas de-*O*-glycosylated PRM did not show any effect on macrophage conidia internalization (**Fig 3**). These results reveal that macrophages recognize and internalize *S*. *prolificans* via PRM, and demonstrate that *O*-glycosylation plays a role in this process.

### Effect of O-glycosylation on the activation of microbicidal mechanisms

**Fig 4:**
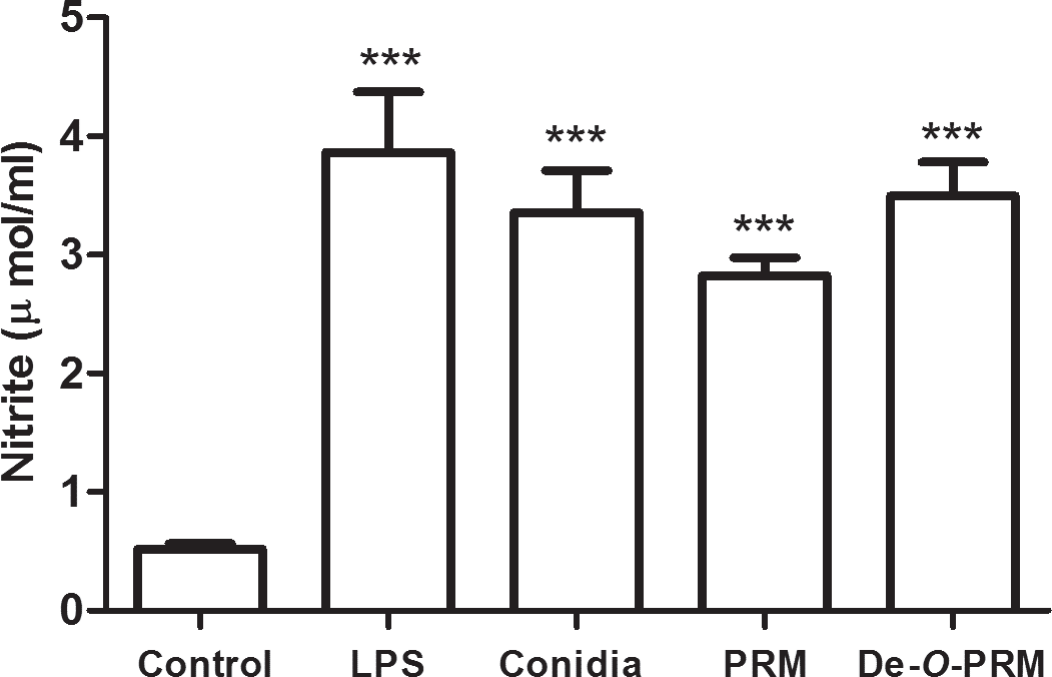
Effect of *O*-glycosylation on the release of nitric oxide (NO). *S*. *prolificans* conidia and PRM led to a significant increase in the levels of NO during interaction with macrophages. *O*-linked oligosaccharides structures were not required for such induction, as observed in the graphs. Values are the means of three independent experiments. Asterisks denote values significantly different from control (*** P < 0.001).

The above results indicate that PRM is recognized by macrophages receptors leading to conidia internalization. Interaction with these receptors could also be responsible for triggering intracellular activation pathways. The activation of phagocytes leads to nitrite production, compounds of primary importance in host defense against microbial pathogens. For assessing the ability of *S*. *prolificans* and PRM to activate macrophage microbicidal mechanisms, conidia, PRM and de-*O*-glycosylated PRM were used to challenge macrophages and the production of NO was quantified by using Griess reagent. As shown in **Fig 4**, macrophages stimulated with *S*. *prolificans* conidia, as well as LPS, had a significant increase in NO levels compared to non-stimulated macrophages. We suggest that the production of these microbicidal components involves the recognition of PRM because macrophages were able to produce NO in response to this molecule at the tested concentration (25 μg/mL) (**Fig 4**). The levels of NO induced by PRM were comparable to those induced by de-*O*-glycosylated pRM, suggesting that *O*-linked oligosaccharides structures are not required for such induction (**Fig 4**).

### Macrophage viability assay

**Fig 5:**
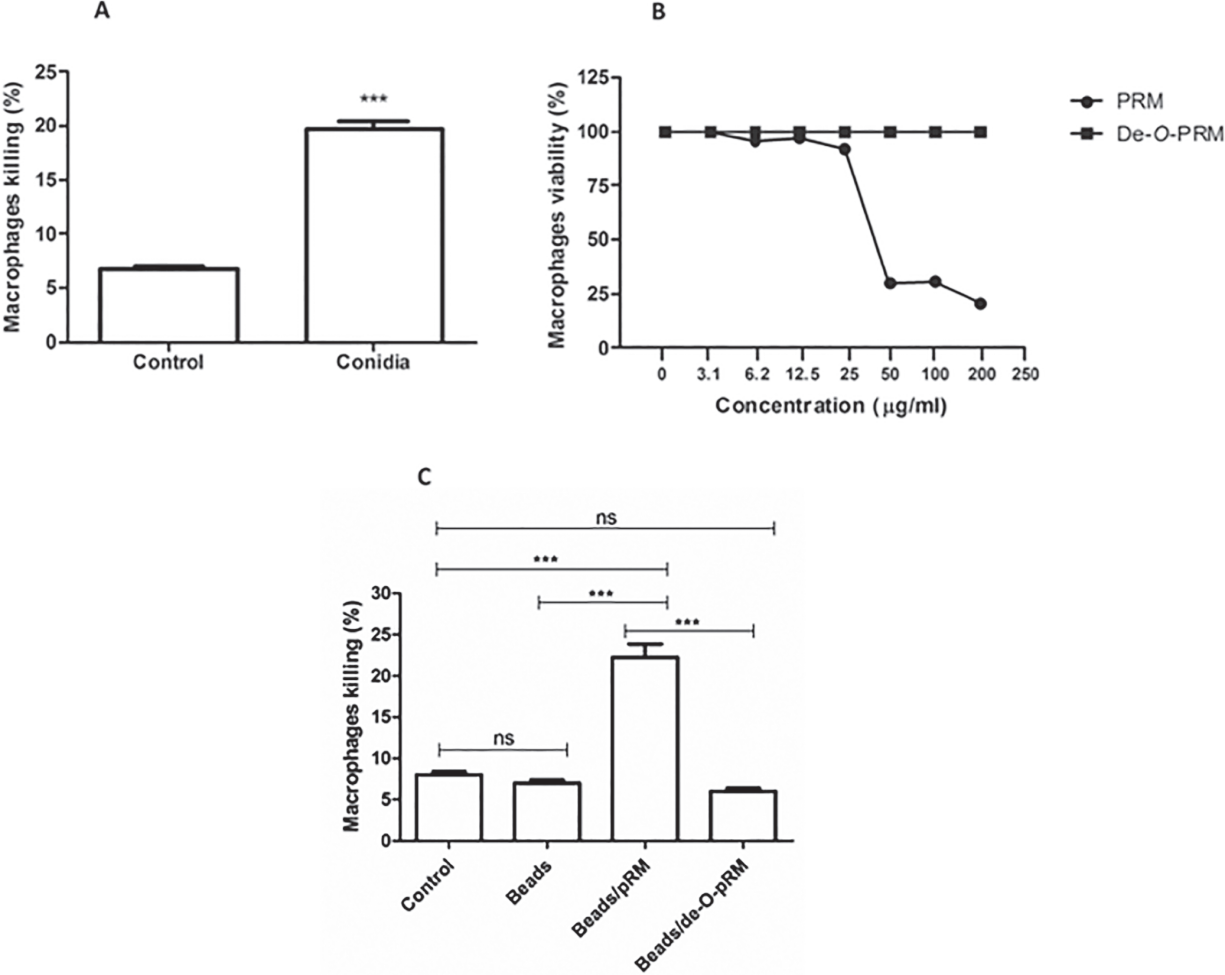
Evaluation of macrophages viability. (**A**) *S*. *prolificans* conidia cells are able to kill macrophages during the first hour of interaction. (**B**) Cytotoxicity of soluble PRM accessed by the neutral red dye-uptake method on macrophages during 24 h of incubation was measured by the absorbance measured at 490 nm in a spectrophotometer. (**C**) Beads coated with PRM and de-*O*-PRM showed that *O*-linked oligosaccharides are responsible for the toxic effects of PRM on macrophages. Values are the means of three independent experiments. Asterisks denote values significantly different from control (* P < 0.05; *** P < 0.001).

To examine *S*. *prolificans* macrophage killing, macrophages were incubated for 1 hour with conidia at the same conditions previously described for the phagocytic assays and their viability was accessed by Trypan blue exclusion. **Fig 5A** shows the percentage of macrophages killed following incubation with *S*. *prolificans* conidia. To further determine the effect of PRM on conidia-induced macrophage killing, the cytotoxicity of soluble PRM was assessed by the neutral red dye-uptake method. A 50% reduction in the number of viable cells was induced by a PRM concentration of 50 μg/ml. Soluble de-*O*-glycosylated PRM did not result in cytotoxicity at concentrations up to 200 μg/ml, the maximum concentration tested (**Fig 5B**). Furthermore, latex beads coated with PRM or de-*O*-glycosylated PRM were used as particulate stimuli for the macrophages using the same ratio of 5:1 used in the phagocytosis assay. The interaction with PRM-coated beads caused a decrease in cell viability, similar to that caused by conidia (**Fig 5C**). However, macrophage viability was unaffected by the beads coated with de-*O*-glycosylated PRM when compared to controls (**Fig 5C)**. These results indicate that the *O*-linked oligosaccharides are responsible for the toxic effects of PRM on macrophages.

### Pro-inflammatory cytokine release induced by S. prolificans PRM require the O-linked chains

**Fig 6:**
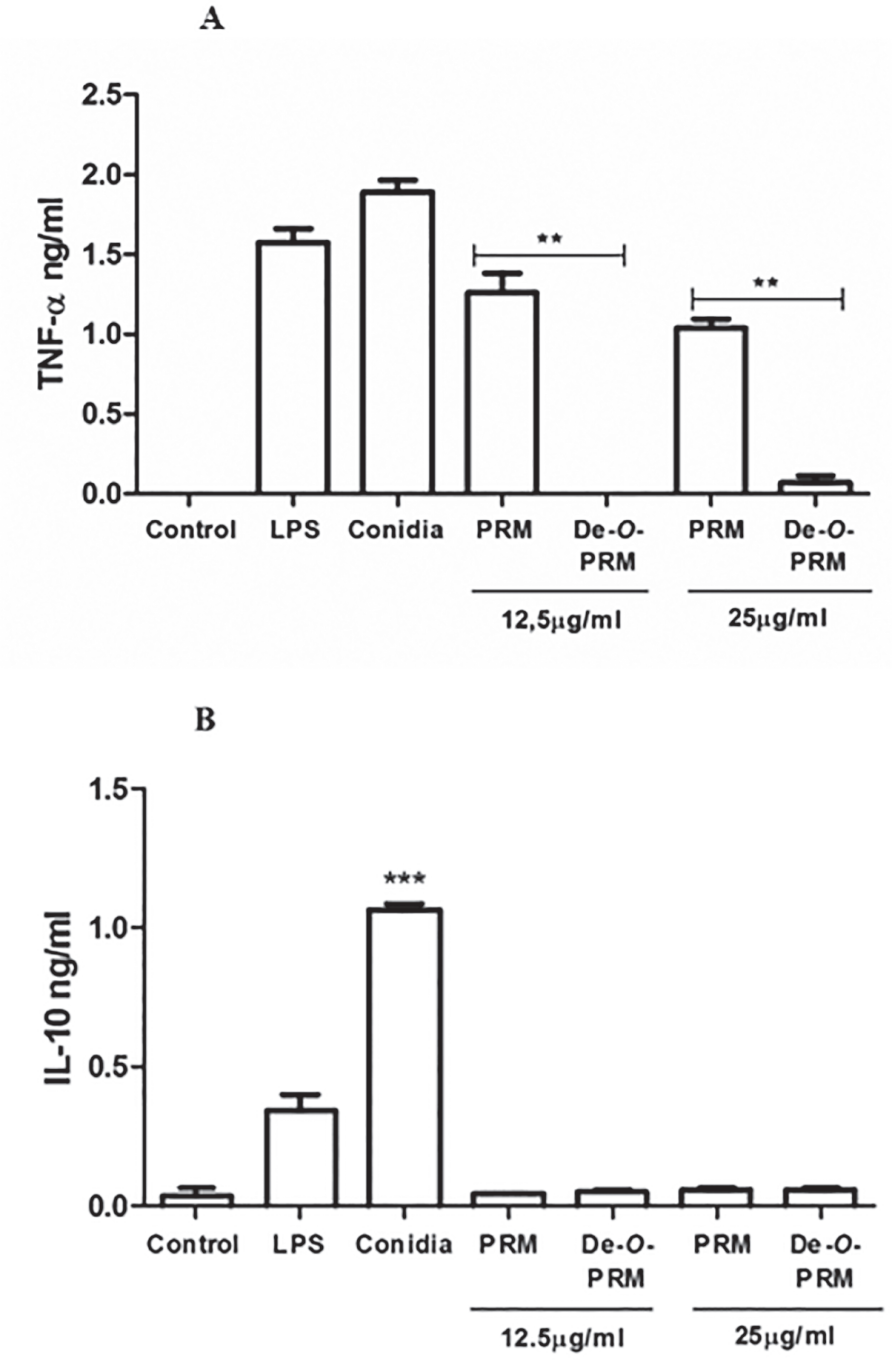
Cytokine release induced by conidia and PRM from *S*. *prolificans*. (**A**) *O*-linked oligosaccharidic chains are crucial to the production of TNF-α. Both *S*. *prolificans* conidia and PRM were able to stimulate the production of TNF-α. This effect was abolished when the *O*-linked oligosaccharides were removed from PRM molecules. (**B**). *S*. *prolificans* conidia were able to stimulate the production of IL-10, but this effect was independent of PRM. Values are the means of three independent experiments. Asterisks denote values significantly different from control (** P = 0.002; *** P < 0.001).

The role PRM plays in *S*. *prolificans* conidia induction of TNF-α, a distinct proinflammatory cytokine, and of the anti-inflammatory IL-10, by macrophages was examined (**Fig 6A and 6B**). The TNF-α cytokine was produced by the macrophages stimulated with conidia and with PRM (**Fig 6A**). When de-*O*-glycosylated PRM was tested, a significant decrease in cytokine levels was observed for TNF-α (**Fig 6A**), suggesting that the *O*-linked oligosaccharidic chains are important moieties for inflammatory response by inducing TNF-α secretion (**Fig 6B**). The IL-10 cytokine was only produced by macrophages when stimulated with conidia, and not when stimulated by PRM or de-*O*-glycosylated PRM (**Fig 6B**).

## Discussion

Glycosylated molecules present in the outer layer of the fungal cell wall are involved in important biological events related to virulence and pathogenicity. Peptidorhamnomannans (PRMs) are common cell wall components that are found in *P*. *boydii* [7], *S*. *apiospermum* [13] and *S*. *prolificans* [11]. These glycoconjugates consist of a peptide chain substituted with both *O*- and *N*-linked glycans. They are structurally distinct, however they present a conserved structural component, α-Rha*p*- (1 → 3)-α-Man*p*-(1 → 2)-α-Man*p*-(1→ [9]. *O*-linked oligosaccharides ranging from tri- to hexasaccharides were isolated from PRM of *P*. *boydii* mycelia and of *S*. *prolificans* mycelia and conidia [5, 11, 13] and their immunodominance was evaluated [5, 7, 18]. In addition to the contribution of *O*-linked oligosaccharides to the antigenicity of PRMs, *O*-glycosylation is critical for fungal adhesion to host cells. De-*O*-glycosylated *P*. *boydii* PRM efficiently inhibited the adhesion of *P*. *boydii* conidia to epithelial cells (HEp2) and prevents endocytosis [18]. *O*-glycosylation of proteins is critical for adhesion and virulence of *Candida albicans* [19]. *N*- and *O*-linked glycans are involved in the recognition, binding and phagocytosis of *C*. *albicans* by human polymorphonuclear cells (PMNs) [20]. In this work, we showed that PRM isolated from the conidia cell wall of *S*. *prolificans* is involved in important aspects of fungal pathogenicity. We demonstrated that *O-*linked oligosaccharides are the key determinants for the phagocytosis of conidia by murine macrophages, fungal macrophage killing, and induction of the inflammatory response.

The inhibition of conidial reactivity with rabbit immune sera by soluble PRM indicates that the glycoprotein is not masked by other structures on the conidia surface, and thus it can mediate conidial interactions with immune cells.

In *C*.*albicans* the loss of cell wall *O*-mannan exposes the inner β-1,3-glucan facilitating the recognition by Dectin-1 and consequentely enhancing phagosome maturation [21].

To demonstrate that conidia internalization depends to a significant extent on PRM recognition and that the *O*-linked chains from PRM are determinants for uptake of *S*. *prolificans* by macrophages, we used the de-*O*-glycosylated PRM (*N*-linked PRM), obtained after removing *O*-linked oligosaccharides by β-elimination. Our results demonstrated that PRM lacking *O*-linked chains was not able to inhibit *S*. *prolificans* phagocytosis, implying that *S*. *prolificans O*-linked chains are recognized by phagocytic receptors.

Several fungi, including *Aspergillus fumigatus* [22], *Penicillium marneffei* [23] and *C*.*albicans* [24, 25], induce macrophage microbicidal functions through the production of toxic reactive oxygen (ROS) and nitrogen species., However, other pathogenic species, such as *Histoplasma capsulatum* [26], *Paracoccidioides brasiliensis* [27], *Coccidioides immitis* [28] and *Blastomyces dermatitidis* [29], evade the immune system by inhibiting these responses. The enzymatic systems devoted to producing ROS and nitrogen species are triggered when cells are activated by the recognition of molecular patterns from invading pathogen (PAMPS), most commonly via pattern recognition receptors (PRRs). Fungal β-glucans have a marked role in activating oxidative bursts, and it is extensively reported to be dependent on Dectin-1 recognition [30–32]. Specific TLR agonists also induce the production of oxygen and nitrogen reactive intermediates and cytokines through MAPK signaling pathways [33, 34]. While the structural and functional characterization of cell wall PAMPS from yeasts, such as *C*. *albicans* and *C*. *neoformans* [35], have been largely investigated, the PAMPs present in filamentous fungi, such as S*cedosporium* spp., are still poorly understood [36]. We found that *S*. *prolificans* conidia have the ability to trigger NO production by macrophages. PRM is involved in this induction, but the *O*-linked oligosaccharide chains are not required for this effect as the de-*O*-glycosylated PRM induced the same amounts of NO as intact PRM.

The large array of oxidative molecules generated by macrophages has potential cytotoxicity against a variety of microorganisms. Filamentous fungi engulfed by phagocytes, if not killed by the microbicidal arsenal of these cells, can germinate to form hyphae, piercing the cells and growing out of them [35]. Our results demonstrated that the *S*. *prolificans* infection promoted macrophage killing. Soluble PRM, as well as PRM-coated beads, were able to induce macrophage death, suggesting that the recognition of PRM exposed on the conidial surface could be mediating this process. Thus, it seems that *S*. *prolificans* PRM can be involved in fungal survival in the host by promoting the evasion of macrophage microbicidal mechanisms. Interestingly, the cytotoxicity of PRMs is lost after *O*-linked oligosaccharides removal, suggesting that these moieties may play an important role in the persistence of the fungus in the host. Despite the fact that monocyte-derived macrophages (MDM) phagocytose *S*. *prolificans* conidia in a manner comparable to the phagocytosis of *A*. *fumigatus*, they inhibit the germination of *S*. *prolificans* conidia less efficiently than they inhibit *A*. *fumigatus* [37]. Thus our results indicate that the *S*. *prolificans* PRMs must represent important mediators for fungal evasion of microbicidal activity of phagocytes, contributing to the pathogenicity of *S*. *prolificans*.

In addition to NO generation, TNF-α is also released by macrophages challenged with *S*. *prolificans* conidia and PRM. Unlike NO production, cytokine production is dependent on the presence of the *O*-linked oligosaccharides from PRM. However, the release of IL-10 was detected only when macrophages were stimulated with conidia and not with PRM. Putative endotoxin contamination was excluded as a possible explanation for this effect by the use of polymyxin B in a dose that abrogated LPS-induced effects. This treatment did not affect the stimulating capacity of conidia or PRM. Our results imply that the differential recognition of PRM motifs by macrophages can lead to the activation of different cellular events. We have previously demonstrated that conidia and *N*-linked rhamnomannans from *P*. *boydii* induced the production of TNF-α and IL-10 cytokines through TLR4 signaling with MAPK phosphorylation [38]. *S*. *prolificans* PRM, on the other hand, is able to stimulate TNF-α but not IL-10. ^13^C NMR and methylation analysis showed significant structural differences between the *O*-linked chains from *S*. *prolificans* and *P*. *boydii* PRM. Such differences consist of a terminal *O*-methylrhamnose replacing the rhamnose capping group, and a pentasaccharide lacking the β-galactopyranosyl side-chain in *S*. *prolificans* oligosaccharides [11]. Distinct patterns of *O*-glycosylation in *P*. *boydii* and *S*. *prolificans* could justify the fact that only *S*. *prolificans O*-linked chains are able to induce TNF-α release. In *C*.*albicans*, *O*-linked mannans induce innate immune activation associated with pro-inflammatory cytokine release via TLR4 [39]. In addition to TLR4, Dectin-2 is a receptor involved in the recognition of *C*. *albicans* α-mannans [40, 41]. Dectin-2 is also the receptor involved in the recognition of the filamentous fungi *Malassezia* spp., and its ligand has been demonstrated to be a glycoprotein containing *O*-linked α-1,2 mannobiose residues [42]. Further studies are necessary to determine the receptor involved in *S*. *prolificans* PRM recognition. With the observation that different cellular events require different PRM motifs, we postulate that the interaction between *S*. *prolicans* conidia and macrophages could involve recognizing PRM by more than one pattern recognition.
